# Efficacy and mechanism of combined treatment with transcranial direct current stimulation and zolpidem for treatment-resistant insomnia: a study protocol for a prospective, double-blind, randomized controlled trial

**DOI:** 10.3389/fpsyt.2026.1743024

**Published:** 2026-02-12

**Authors:** Yudong Wang, Jian Liu, Shanshan Cheng, Jun-Li Cao, Jianyou Zhang, Luo Zhang

**Affiliations:** 1 Department of Anesthesiology, The Affiliated Hospital of Yangzhou University, Yangzhou, China; 2 Yangzhou University Faculty of Medicine, Yangzhou, China; 3 Department of Anesthesiology, The First People’s Hospital of Guannan County, Lianyungang, China; 4 Department of Anesthesiology, The Affiliated Hospital of Xuzhou Medical University, Xuzhou, China; 5 National Medical Products Administration Key Laboratory for Research and Evaluation of Narcotic and Psychotropic Drugs, Xuzhou Medical University, Xuzhou, China; 6 Jiangsu Province Key Laboratory of Anesthesiology, Xuzhou Medical University, Xuzhou, China; 7 Jiangsu Key Laboratory of Applied Technology of Anesthesia and Analgesia, Xuzhou Medical University, Xuzhou, China

**Keywords:** combined therapy, functional magnetic resonance imaging, transcranial direct current stimulation, treatment-resistant insomnia, zolpidem

## Abstract

**Background:**

Treatment-resistant insomnia remains a major unmet clinical challenge, as a substantial proportion of patients fail to achieve long-term remission with cognitive behavioral therapy or pharmacotherapy alone. Transcranial direct current stimulation (tDCS) has shown promise in modulating cortical excitability and improving sleep quality through non-invasive neuromodulation, whereas zolpidem (ZOL), a GABA-A receptor agonist, provides rapid but transient symptomatic relief. However, whether their combination offers additive therapeutic benefits and how such effects are represented at the neural level remain unknown.

**Methods:**

This prospective, double-blind, randomized controlled trial will enroll 165 patients with treatment-resistant insomnia. Participants will be randomly assigned (1:1:1) to one of three groups: (A) active tDCS + ZOL, (B) active tDCS + placebo, and (C) sham tDCS + ZOL. The intervention lasts four weeks, with 20 tDCS sessions (2 mA, 20 min/day, 5 days/week, anode over left and cathode over right dorsolateral prefrontal cortex) and nightly oral administration of ZOL or placebo. The primary outcome is the response rate at week 4, defined as the percentage of those having at least a 50% reduction in insomnia symptoms from baseline as measured via the Pittsburgh Sleep Quality Index (PSQI). Secondary outcomes include response rates at 8 and 12 weeks, clinical remission (PSQI<5), changes in PSQI and Insomnia Severity Index scores, sleep architecture monitored by a wearable device, and mood assessments using Hamilton Depression Rating Scale and Hamilton Anxiety Rating Scale. Resting-state functional MRI (rs-fMRI) will be acquired at baseline and 4 weeks to explore alterations in regional brain activity and functional connectivity.

**Discussion:**

This trial will systematically evaluate the efficacy and neurobiological mechanisms of tDCS combined with zolpidem in treatment-resistant insomnia. By integrating subjective clinical assessments, objective digital sleep monitoring, and neuroimaging biomarkers, it aims to elucidate whether these combined pharmacological and neuromodulatory interventions produce additive effects. The findings are anticipated to establish a mechanistic foundation for personalized, multimodal sleep therapeutics, thereby potentially advancing the management paradigm for treatment-resistant insomnia.

**Clinical Trial Registration:**

https://www.chictr.org.cn/showproj.html?proj=288195, identifier ChiCTR2500111601.

## 1 Introduction

Insomnia disorder (ID) is one of the most common sleep disorders, with a prevalence rate of approximately 10 to 20% (1). It significantly affects an individual’s physical and mental health, reduces work efficiency and alertness, impairs quality of life, and increases the economic burden (2–4).

Current first-line treatments include cognitive behavior therapy (CBT) and hypnotic drugs (5), but not all patients achieve long-term remission to them (6). Outcome assessments of randomized clinical trials involving CBT, pharmacological agents, or both, demonstrate a clinically meaningful response rate between 50 and 75%; however, these studies rarely extend beyond 12 months (6). At least a quarter of these patients experience a relapse within the first year (7). Further, most of the clinical trials exclude patients with severe comorbid conditions or who have failed prior therapy (6). Additionally, 40-92% of these patients have comorbid psychiatric conditions such as anxiety or depression, forming a vicious cycle of “insomnia-mood disorders” (8). This treatment-resistant insomnia involves multiple physiological, psychological, and environmental factors, making it difficult to achieve sustained and effective symptom control through a single therapeutic approach (9). Consequently, there is a pressing need for safe and effective multimodal treatment programmers for insomnia.

Transcranial direct current stimulation (tDCS) is a non-invasive cranial electrical stimulation consisting of a constant low-intensity electrical current applied to the scalp to modulate brain activity. Weak direct currents can penetrate the cranium, modulating the amplitude and orientation of the intracranial electric field, thereby inducing excitatory alterations in the cerebral cortex (10). Anodal stimulation triggers neuron depolarization, increasing cortical excitability, while cathodal stimulation triggers neuron hyperpolarization, decreasing cortical excitability (11, 12). Evidence from multiple studies demonstrates that insomnia patients receiving tDCS therapy show improved subjective sleep quality, as measured by declines in Pittsburgh Sleep Quality Index (PSQI) scores (13–15). A 12-week trial documented significant enhancements in patients’ sleep quality (15). Beyond symptomatic relief, a mechanistic investigation combining tDCS with electroencephalography demonstrated that slow-wave oscillatory stimulation synchronises brain waves with the sleep’s slow-wave frequency in insomnia patients, potentially facilitating sustained sleep stabilization (16). tDCS exerts a lasting “root-cause” therapeutic effect through long-term neuromodulation, but it acts slowly (17). On the other hand, zolpidem (ZOL) is a short-acting non-benzodiazepine hypnotic that selectively targets GABA-A receptors and rapidly induces sleep (18). In contrast to tDCS, zolpidem provides rapid relief for “difficulty falling asleep,” but at the cost of increased risks with long-term use (19). Given that both have their advantages, combining neuromodulation with pharmacotherapy represents a promising clinical strategy.

The effect of combining tDCS and zolpidem for treatment-resistant insomnia remains unclear. Specifically, whether this combination is superior to a single-modality neurotherapy or medication use requires further investigation. Therefore, we will perform a double-blind, randomised, parallel-group, controlled trial using tDCS combined with zolpidem to systematically evaluate the efficacy and enhancement over single-modality treatment of combining tDCS with zolpidem in patients with treatment-resistant insomnia. Additionally, we intend to integrate resting-state functional magnetic resonance imaging (rs-fMRI) to explore the underlying neural mechanisms in depth from the perspectives of regional brain activity and functional network connectivity. We anticipate that this study will contribute to sleep medicine by providing a more effective and safer therapeutic option for treatment-resistant insomnia, potentially establishing a new treatment protocol and guiding clinicians in selecting the most efficacious strategies compared to traditional single-modality therapies.

## 2 Materials and methods

### 2.1 Study design and patients

**Figure 1:**
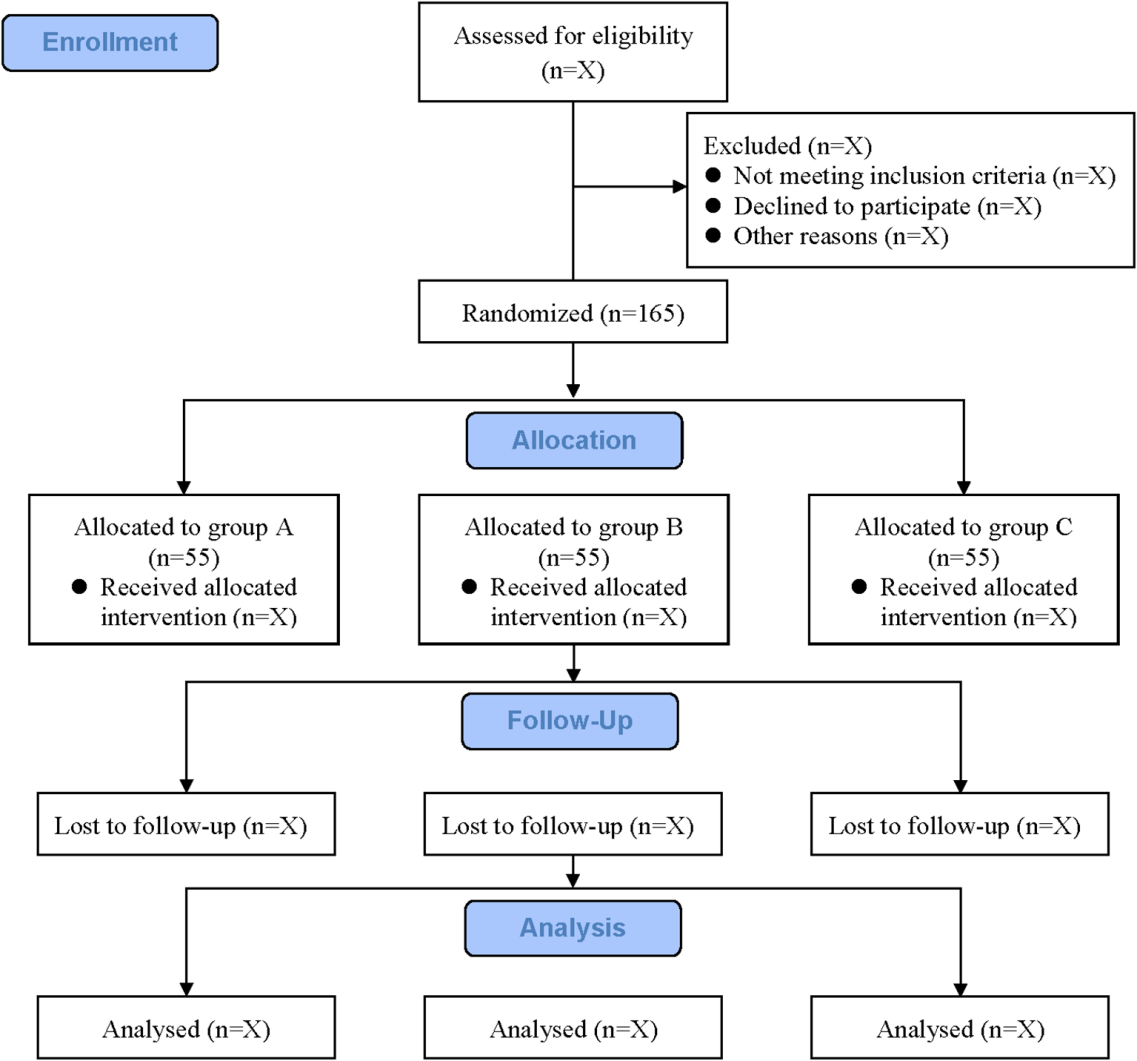
Study flow diagram. Grouping according to modes of intervention. Group A, active tDCS + ZOL; Group B, active tDCS + placebo; Group C, sham tDCS + ZOL.

This clinical trial was designed as a prospective, double-blind, randomized controlled study and will be conducted at the Affiliated Hospital of Yangzhou University. A total of 165 patients will be recruited based on stringent inclusion and exclusion criteria. All participants will provide written informed consent before enrollment (**Supplementary Material 1**). The study adheres to the Standard Protocol Items: Recommendations for Interventional Trials (SPIRIT) guidelines (20). **Table 1** shows the SPIRIT schedule of enrolment, intervention, and assessments. The study flow diagram is shown in **Figure 1**.

**Table 1:** Study schedule of patient enrolment, study interventions and outcome assessment.

Study period						
Time point	Enrollment	Allocation	Baseline	Treatment	Follow-up	
	-2 day	-1 day	0	4 weeks	8 weeks	12 weeks
Enrollment						
Inclusion criteria	☑					
Exclusion criteria	☑					
Informed consent	☑					
Randomization and allocation		☑				
Interventions						
Active tDCS + ZOL group	1–4 weeks					
Active tDCS + placebo group	1–4 weeks					
Sham tDCS + ZOL group	1–4 weeks					
Outcome assessment						
PSQI			☑	☑	☑	☑
ISI			☑	☑	☑	☑
Sleep wristband monitoring			☑	☑	☑	☑
HAMA			☑	☑	☑	☑
HAMD			☑	☑	☑	☑
Rs-fMRI examination			☑	☑		
Adverse events				☑	☑	☑

According to SPIRIT 2013 statement: defining standard protocol items for clinical trials.

tDCS, transcranial direct current stimulation; ZOL, zolpidem; PSQI, Pittsburgh Sleep Quality Index; ISI, Insomnia Severity Index; HAMA, Hamilton Anxiety Rating Scale; HAMD, Hamilton Depression Rating Scale; rs-fMRI, resting-state functional magnetic resonance imaging

### 2.2 Inclusion criteria

1. Age 18–75 years;
2. Diagnosis of chronic insomnia disorder according to the International Classification of Sleep Disorders-third edition (ICSD−3);
3. Insomnia lasting ≥6 months, and meeting either of the following criteria: a) an inadequate response (a PSQI score >7) to both at least one full course of cognitive behavioral therapy for insomnia (CBT-I) and at least one pharmacological treatment for ≥3 months; b) an inadequate response (a PSQI score >7) after pharmacological treatment with at least two types of medications with distinct mechanisms of action for ≥3 months;
4. Voluntary participation in this study and signed informed consent;
5. Agreeing not to take other pharmacological or nonpharmacological treatment during the whole trial.

### 2.3 Exclusion criteria

1. Craniocerebral or scalp injury;
2. Combine severe neurological or mental disorders;
3. Doing night shift work;
4. Prior exposure to electrocon vulsive therapy, transcranial magnetic stimulation, or transcranial current stimulation;
5. With any metal implants in the body, claustrophobia, or any other reasons that would prevent an MRI
6. Being pregnant or lactating;
7. Known hypersensitivity to trial-related medications;
8. History of drug or alcohol abuse/dependence;
9. Participation in concurrent clinical trials.

### 2.4 Withdrawal criteria

1. Voluntary withdrawal;
2. Protocol violation;
3. Concomitant medications or therapies interfering with trial outcomes;
4. Severe adverse events or complications, making further treatment inappropriate;
5. Loss to follow-up.

### 2.5 Randomization and blinding

Study participants will be randomly allocated to one of three groups (group A: active tDCS + ZOL, group B: active tDCS + placebo, group C: sham tDCS + ZOL) in a 1:1:1 ratio based on computer-generated random numbers made by an independent research staff who is neither involved in the enrolment nor in the assessment of the participants. Each participant will receive a unique, de-identified alphanumeric study code that records their enrollment order and allocation group, ensuring data traceability and confidentiality. The randomization assignments will be safeguarded in sequentially numbered, opaque, sealed envelopes by a designated research coordinator. Immediately before the patient enters the treatment room, a nurse will unseal the envelope to confirm the assigned group. After verifying the details of group allocation, the envelope will be resealed, and this step will be carried out without revealing the information to the patients. To maintain blinding, participants will be advised during the informed consent process that possible sensory perceptions (such as pricking sensations, needle-like stimulation, warmth, and vibration) may occur during treatment and that their intensity will be independent of therapeutic effectiveness. Additionally, all stimulation devices are identical in appearance, with differences restricted to electrical output parameters, and the ZOL and placebo pills have the same size, appearance, weight, and odor. This matching ensures the integrity of the blinding procedure. Blinding success will be evaluated once, at the end of the 4-week intervention period, using a standardized questionnaire for both participants and intervention providers. Participants and intervention providers will be asked to guess their own group assignment and to rate their confidence in that guess. Throughout the study, all outcome assessors, data analysts, intervention providers, and study participants will remain blinded to group allocation. Outcome assessors will additionally undergo rigorous blinding training prior to the study commencement to standardize assessment procedures and minimize subjective bias.

### 2.6 Emergency unblinding

Should a serious adverse event (SAE) or other emergency occur during the trial, immediate unblinding will be performed to enable appropriate medical intervention. The decision will be a collaborative one between the principal investigator (PI) and the attending physician. Each unblinding event will be fully documented in the Case Report Form (CRF), including the reason for unblinding, date, time, personnel involved, and subsequent actions taken.

### 2.7 Study interventions

The active tDCS + ZOL (combined) group will be treated with tDCS using a tDCS instrument and take 10 mg zolpidem 10–30 minutes before bedtime nightly. The stimulator model is E-TDCS03 (Shenzhen Ailite Medical Technology Co., Ltd., Shenzhen, China), which provides 2 mA of stable direct current power through a battery. Two sponges (5 × 5 cm², soaked in 0.9% normal saline to ensure conductivity) will be placed on the rubber electrodes for stimulation. The left (F3) and right (F4) dorsolateral prefrontal cortex (DLPFC) regions, as defined by the International 10–20 EEG Electrode Distribution System (21), will be the sites for the anode and cathode electrode placement, respectively. A fade-in/fade-out design (30 s each) will be used to decrease potential skin sensations during the beginning and end of the stimulation (22). During tDCS intervention sessions, patients will be requested to sit in a comfortable reclining chair, switch off their smartphones, and are encouraged to hydrate, relax, or, and fall asleep. Communication with nursing staff will be minimized. The tDCS intervention will be administered according to a fixed protocol: 20-minute sessions, a fixed time during the daytime once daily from Monday through Friday, over four consecutive weeks. This regimen will be delivered by two trained nurses.

The active tDCS + placebo group will be treated with tDCS and take a placebo pill 10–30 minutes before bedtime nightly. The stimulation modality, stimulation electrode positions, stimulation region, and stimulation duration will match those adopted in the combined group.

The sham tDCS + ZOL group will undergo treatment with pseudo-stimulation using tDCS and take 10 mg zolpidem 10–30 minutes before bedtime nightly. The stimulation modality, stimulation electrode positions, stimulation region, and stimulation duration will be identical to those applied in the combined group. The pseudo-stimulation will only output a current of up to 2 mA for 10 seconds at the start and end of the session, allowing participants to perceive a sensation comparable to real stimulation. The device will cease delivering electrical stimulation throughout the middle of the 20-minute session.

During the entire intervention period, researchers with professional training will strictly abide by the protocol and closely track participants’ responses to prevent adverse reactions. Should participants report unbearable discomfort, the intervention will be terminated immediately.

### 2.8 Primary outcome

The primary outcome measure is the response rate at 4-week follow-up (time point of finishing 4-weeks treatment period), defined as the percentage of those having at least a 50% reduction in insomnia symptoms from baseline as measured via the PSQI (9). The PSQI is a 19-item questionnaire comprising seven components: sleep quality, sleep latency, sleep duration, sleep efficiency, sleep disturbance, use of sleep medication, and daytime dysfunction. Each component is rated from 0 to 3, yielding a global score between 0 and 21, where a higher total score denotes worse sleep quality. This scale is widely employed to assess participants’ subjective sleep quality over the preceding month (5, 23).

### 2.9 Secondary outcomes

1. The response rate at 8-week follow-up and 12-week follow-up.
2. Clinical remission at baseline, 4-week follow-up, 8-week follow-up and 12-week follow-up, defined as a PSQI total score < 5 (24).
3. Changes in other sleep parameters measured via the PSQI, i.e., total score, sleep onset latency, total sleep time, sleep efficiency, sleep quality, and daily disturbances.
4. The severity of the patients’ insomnia at baseline, 4-week follow-up, 8-week follow-up and 12-week follow-up will be assessed using the Insomnia Severity Index (ISI), which consists of seven items measuring day and night symptoms of insomnia in individuals. The ISI includes questions on perceived difficulty (Item 1), falling asleep (Item 2), time of awakening (Item 3), satisfaction with current sleep pattern (Item 4), interference with daily functioning (Item 5), noticeability of the impact of lack of sleep on others (Item 6), and the degree of perceived distress or concern caused by the sleep problem (Item 7). Subjects rated each question on a 5-point Likert scale (0-4), and the total score ranges from 0 to 28, with higher scores indicating more severe insomnia (25).
5. Patient sleep patterns, including deep sleep, light sleep, rapid eye movement (REM) sleep, and awake time, will be monitored using the wearable device (Fitbit Inspire 3, Fitbit Inc) at baseline, 4-week follow-up, 8-week follow-up and 12-week follow-up.
6. At baseline, 4-week follow-up, 8-week follow-up and 12-week follow-up, patients’ depression and anxiety will be assessed by Hamilton Depression Scale (HAMD) and Hamilton Anxiety Scale (HAMA) total scores, respectively, as well as each factor score. A 17-item version of the HAMD will be used, with each item involving different aspects of depression symptoms, including four components: core symptoms (items 1-3), insomnia (items 4-6), anxiety (items 9-13, 15), and somatic factors (items 7, 8, 14, 16, 17). Each item is rated on a 0–4 point scale, with a total score of ≤ 7 deemed indicative of the absence of depressive symptoms. The HAMD evaluates the severity of participants’ depressive symptoms over the past 1–2 weeks, where items 4–6 focus on assessing sleep quality (26). The HAMA scale includes 14 items, each of which involves different aspects of anxiety symptoms, including two components: psychic anxiety (items 1-6, 14) and somatic anxiety (items 7-13). Each of its items is rated on a 0–4 scale, and a total score ≤ 7 serves as the cut-off for indicating no anxiety symptoms. The HAMA is employed to evaluate the severity of participants’ anxiety symptoms over the past 1–2 weeks (27).
7. Resting-state fMRI data will be collected at baseline and 4-week follow-up to analyze alterations in brain activity (assessed via fractional Amplitude of Low-Frequency Fluctuations [fALFF] and Regional Homogeneity [ReHo]) and functional connectivity [FC]).
8. Incidence of adverse events, evaluated by treatment-emergent adverse event.

### 2.10 Adverse events and safety

Potential adverse events (AEs) related to electrical stimulation, including skin redness, pruritus, headache, epileptic seizure, hypomania, and common adverse events such as dizziness, or nausea, will be closely monitored. Any AE will trigger a prompt clinical evaluation and necessary medical care. Mild AEs (e.g., minor discomfort) will be managed with localized symptomatic treatment. In cases of moderate or severe AEs (e.g., syncope, severe pain), the intervention will be immediately halted or discontinued, and the participant will be continuously monitored by qualified medical personnel until complete resolution. Comprehensive emergency protocols are established to ensure unwavering patient safety throughout the trial. All AEs will be systematically recorded in the CRF with a comprehensive assessment of their causal relationships.

### 2.11 Sample size calculation

Sample size was calculated using PASS 15.0 software. Response rates at the 4-week follow-up were 53.3% (8/15) in the active tDCS + ZOL group, 20% (3/15) in the active tDCS + placebo group, and 13.3% (2/15) in the sham tDCS + ZOL group in our pilot study. We conservatively assumed a 50% response rate at the 4-week follow-up in the active tDCS + ZOL group, a 20% response rate in the active tDCS + placebo group, and a 15% response rate in the sham tDCS + ZOL group, thus a sample size of 44 per group is required to have a power of 80% and a two-tailed alpha level of 5%. Considering the 20% attrition rate and a block size of 3, we set our enrolment target at 165 subjects (55 per group).

### 2.12 Statistical analysis

Data analyses will be performed using SPSS 26.0 (IBM, Armonk, NY, USA) and GraphPad Prism 10.0 (GraphPad Software, San Diego, CA, USA). The normality of continuous data will be assessed using the Kolmogorov-Smirnov test, and the homogeneity of variances will be examined with Levene’s test. Continuous variables will be presented as mean ± standard deviation if normally distributed, and as median with interquartile range (IQR) otherwise. Between-group differences will be evaluated using one-way ANOVA for normally distributed continuous variables, the Kruskal-Wallis test for non-normally distributed continuous variables. Chi-squared test will be used to evaluate the between-group differences among the three groups in categorical variables. Time-to-event outcomes will be assessed using Kaplan-Meier curves and Log-rank test. Multivariate analyses will utilize multiple linear or logistic regression models based on dependent variable type. To analyze treatment effects, repeated measures data will be analyzed using generalized estimating equations (GEE). This analysis will explore the changes in clinical outcome data across different treatment groups and timepoints. The treatment-by-time interaction will be tested first. If this interaction is statistically significant, between-group differences will be assessed at each individual time point with a Bonferroni correction for multiple comparisons. Otherwise, the main effect of treatment will be tested without applying the Bonferroni correction. The primary analysis will follow the intention-to-treat (ITT) principle, with missing data handled by multiple imputation. A per-protocol analysis will be performed as a sensitivity analysis. A two-sided *p*-value < 0.05 will be considered statistically significant for all tests.

### 2.13 Functional MRI data acquisition and processing

The rs-fMRI images will be acquired using a 3.0 T Ingenia MR scanner from Philips in Amsterdam, Netherlands, equipped with a 32-channel birdcage head coil. All participants will undergo fMRI scanning once before the start of treatment and again at the completion of the 4-week treatment period. During scanning, participants will be instructed to remain motionless and awake, with their eyes closed and minds clear of any thoughts. Functional images will be acquired with the following parameters: field of view (FOV) = 240 × 240 mm, matrix = 64 × 64, repetition time (TR) = 2000 ms, echo time (TE) = 30 ms, slice thickness = 3.5 mm, interslice gap = 0.25 mm, 38 axial slices in parallel, and 240 time points. High-resolution T1-weighted anatomical images will also be captured using the parameters: TR = 10 ms, TE = 4.7 ms, flip angle = 9°, FOV = 256 mm × 240 mm, matrix = 320 × 300, and slice thickness = 0.8 mm.

The fMRI data will be preprocessed using MATLAB-based CONN 18b toolbox within SPM 12. Preprocessing of the whole brain will include correction of slice timing, image realignment, structural segmentation, spatial and functional normalization to a standard MNI space, resampling into 2×2×2 mm voxels, and application of a 6 mm full-width half-maximum (FWHM) Gaussian kernel smoothing. Subjects will be excluded from the data analysis if their mean framewise displacement (FD) exceeds 0.2 mm, to reduce the impact of head motion. The ART feature from the CONN toolbox will be utilized (https://www.nitrc.org/projects/artifact_detect/) to identify outlier time points for movement parameters and overall signal intensity. Potential variables like white matter, cerebrospinal fluid signal, linear trend, subject motion (six rotational/translational motion parameters and six first-order time derivatives), and outliers will be included as regressors in the linear regression analysis. Next, the remaining blood oxygenation level dependent (BOLD) signal will be processed using a band-pass filter (0.008-0.09 Hz) to reduce the impact of low-frequency fluctuations and high-frequency interference.

Following preprocessing, fALFF and ReHo maps will be calculated for each participant. To standardize these metrics for group-level comparison, individual fALFF and ReHo maps will be converted to z-scores by subtracting the global mean and dividing by the global standard deviation. Seed-based FC analysis will be performed using CONN 18b. The left and right dorsolateral prefrontal cortex (DLPFC) will be defined as spherical regions of interest (ROIs) based on probabilistic maps from the SPM Anatomy toolbox (28). At the first-level (individual) analysis, the mean BOLD time series will be extracted from each of the bilateral DLPFC seeds for every participant. Subsequently, Pearson’s correlation coefficients will be computed between each seed’s time series and the time series of all other voxels in the brain, generating individual-level whole-brain correlation maps for each seed. These correlation maps will then be converted to Z-score maps using Fisher’s z-transformation for subsequent group-level analysis. At the second-level (group) analysis, the z-standardized fALFF maps, ReHo maps, and FC Z-score maps will be analyzed using GEE. Statistical significance will be assessed using a cluster-forming threshold of *p* < 0.001 at the voxel level, with cluster-level inferences corrected for family-wise error (FWE) at *p* < 0.05.

## 3 Discussion

This randomized, double-blind, controlled clinical trial is designed to evaluate the efficacy and underlying neural mechanisms of combining tDCS with zolpidem in patients with treatment-resistant insomnia. The study aims to determine whether the combined intervention can provide superior therapeutic benefits compared with either monotherapy and to elucidate the neurobiological substrates of this additive effect using fMRI. By integrating pharmacological and neuromodulatory approaches, this protocol seeks to establish a novel multimodal therapeutic paradigm for treatment-resistant insomnia.

The brain stimulation technique tDCS has emerged as a promising non-invasive neuromodulation technique capable of regulating cortical excitability and modulating dysfunctional neural networks associated with sleep-wake regulation (29). Previous studies of tDCS monotherapy have shown improvements in subjective sleep quality. For example, Zhou et al. and Amedeo et al. observed reductions in PSQI scores and sleep onset latency (13, 30). Zolpidem, on the other hand, acts rapidly through selective agonism of the GABA-A receptor complex to induce sleep, but its long-term efficacy is hampered by tolerance and dependence risks (17). We hypothesize that combining tDCS and zolpidem may offer additive effects—tDCS providing a sustained neuroplastic benefit, and zolpidem offering immediate symptom relief. Although combination therapy is theoretically appealing, it lacks high-grade evidence from rigorously designed randomized controlled trials.

The DLPFC plays a central role in the cognitive-emotional regulation of sleep and interacts with subcortical structures such as the hypothalamus and thalamus (31). Dysfunctional prefrontal-limbic connectivity has been implicated in hyperarousal and impaired sleep initiation (32). Anodal stimulation of the left DLPFC via tDCS is expected to enhance cortical excitability, normalize aberrant neural activity, and modulate top-down inhibitory control over arousal systems (29). Simultaneously, zolpidem’s potentiation of GABAergic neurotransmission may facilitate the synchronization of sleep-related oscillations, particularly in the slow-wave frequency range, thus enhancing restorative sleep (17). Functional MRI metrics—such as fALFF, ReHo, and seed-based functional connectivity—will provide insight into whether this combined therapy induces neuroplastic remodeling within prefrontal and thalami-cortical networks, potentially leading to more stable and efficient sleep architecture. Such mechanistic insights are crucial for future personalized treatment (e.g., selecting regimens for patients with specific neurofunctional phenotypes) and the development of novel therapeutic targets.

This study addresses gaps in previous sleep research by (1) incorporating objective neuroimaging biomarkers to link clinical improvement with regional brain activity and functional network connectivity (2); focusing on treatment-resistant insomnia, a population often excluded from prior trials; and (3) integrating multimodal assessment—including PSQI, ISI, HAMD, HAMA, and wearable sleep monitoring—further enhances the study’s robustness and clinical translatability. If the treatment efficacy is validated, this combined therapy could redefine current treatment algorithms for treatment-resistant insomnia by introducing a personalized, mechanism-based approach. The immediate pharmacological action of zolpidem could alleviate acute symptoms and enhance compliance, while the neuromodulatory effects of tDCS could provide long-term stabilization of neural circuits. Such a dual mechanism may prevent relapse and reduce reliance on sedative-hypnotics. From a translational perspective, elucidating the neural correlates of treatment response could identify imaging biomarkers predictive of therapeutic efficacy, thereby supporting precision sleep medicine.

Several design elements enhance the methodological rigor of this study. First, the randomized, double-blind, parallel-controlled structure minimizes allocation and observer bias. Second, the use of both subjective (PSQI, ISI) and objective (fMRI, wearable device) outcomes allows multidimensional evaluation of sleep improvement. Third, the sample size calculation is based on pilot data with appropriate consideration for attrition, ensuring adequate statistical power. Fourth, adherence to SPIRIT guidelines and strict safety monitoring ensures compliance with ethical standards and clinical trial quality. Together, these measures strengthen the internal validity and reproducibility of findings.

Despite efforts to improve the validity of this study, there are some limitations. First, the single-center design may limit the generalizability of the results, and multi-center validation is needed. Second, while tDCS is safe and well-tolerated, individual variability in scalp resistance and cortical anatomy may affect stimulation efficacy, potentially introducing heterogeneity in response. Third, although wearable devices have seen increasing accuracy in sleep stage classification, their performance remains inferior to the gold standard of polysomnography. Fourth, although fMRI can reveal correlations, causal relationships between neural changes and clinical improvement cannot be fully established. Finally, as this study employs a three-arm rather than a full 2×2 factorial design, it can determine the superiority of the combined therapy but cannot formally test for a synergistic (interactive) effect between tDCS and zolpidem.

Future studies may expand upon this protocol by exploring different stimulation parameters (e.g., current intensity, duration, and electrode montage) or alternative pharmacological combinations targeting specific neurotransmitter systems such as melatonin or orexin pathways. Moreover, machine learning-based analysis of multimodal neuroimaging and clinical data could facilitate the development of individualized treatment algorithms predicting response profiles to combined neurostimulation-drug interventions.

This study protocol proposes a novel, scientifically grounded approach to treating treatment-resistant insomnia by integrating non-invasive brain stimulation with pharmacological therapy. By systematically assessing clinical efficacy and underlying neural mechanisms, it is expected to generate high-quality evidence supporting the additive efficacy of tDCS and zolpidem. The findings may bridge the gap between neurophysiological understanding and therapeutic application, advancing the field of sleep medicine toward more effective, personalized, and mechanism-based treatment strategies.
