# Supplementary material for: Efficacy and mechanism of combined treatment with transcranial direct current stimulation and zolpidem for treatment-resistant insomnia: a study protocol for a prospective, double-blind, randomized controlled trial
Source: Front Psychiatry. 2026 Feb 12;17:1743024. doi: 10.3389/fpsyt.2026.1743024 (PMC12935941; doi:10.3389/fpsyt.2026.1743024)
Supplement: Supplementary file 1 [file SupplementaryFile1.docx]

Informed consent

Dear Sir/Madam:

We sincerely invite you to participate in a clinical study titled “Efficacy and mechanism of simultaneous combining of transcranial direct current stimulation and zolpidem on patients with intractable insomnia". Before you decide whether to participate in this study, please read the following carefully to help you understand the study's purpose, process and duration, as well as the potential benefits, risks and inconveniences that may result from your participation. If you have any questions or do not understand something, please consult the researcher (doctor) until you are fully clear. You can also discuss with your family, relatives, friends, etc. to help you make the decision that best suits your interests.

The following is an introduction to the study: I. Background and purpose of the research

With the accelerated pace of life and increased mental stress in modern society, the incidence of intractable insomnia has been on the rise year by year. Approximately 10%–15% of insomnia patients respond poorly to conventional first-line treatment and have a disease duration exceeding 3 months. Currently, long-term use of benzodiazepines is associated with dependence and cognitive impairment; while cognitive behavioral therapy (CBT) has fewer side effects, it exhibits significant individual differences in efficacy and insufficient patient adherence.

Transcranial direct current stimulation (tDCS) can regulate cortical excitability and improve neural plasticity, whereas zolpidem enhances inhibitory transmission in the central nervous system. This study aims to systematically evaluate the efficacy and safety of tDCS combined with zolpidem versus tDCS alone and zolpidem alone in the treatment of intractable insomnia through a three-arm randomized double-blind controlled trial. Additionally, it will integrate resting-state functional magnetic resonance imaging (rs-fMRI) to explore the underlying neural mechanisms in depth, from the perspectives of local brain activity and functional network connectivity.

II. Specific procedures and procedures

According to the inclusion and exclusion criteria, eligible and willing study participants were screened, provided their study details, voluntarily selected and signed informed consent forms, checked basic information, recorded relevant medical history. Based on randomization outcomes, patients in the combined treatment group will receive active tDCS at 2 mA once daily at fixed times. The treatment course consists of 5 consecutive sessions followed by 2-day breaks over 4 weeks (20 sessions total), concurrently with 10 mg zolpidem taken 10-30 minutes before bedtime nightly. The zolpidem group will receive sham tDCS along with 10 mg zolpidem 10-30 minutes before bedtime nightly. The tDCS group will receive active tDCS with a matching placebo pill taken 10-30 minutes before bedtime nightly.

The Pittsburgh Sleep Quality Index (PSQI) and Insomnia Severity Index (ISI) will be administered at baseline, 4, 8, and 12 weeks to evaluate overall sleep quality. Sleep parameters including sleep latency and sleep architecture, along with other physiological measures, will be continuously monitored using smart wearable devices at baseline, 4, 8, and 12 weeks. Emotional states will be assessed using the Hamilton Anxiety Scale (HAMA) and Hamilton Depression Scale (HAMD) at baseline, 4, 8, and 12 weeks. Resting-state functional MRI will be performed at baseline and upon completion of the 4-week intervention.

III. What do you need to do if you participate in the study

1. You need to understand the specific situation of this study in detail, under the

premise of full understanding, voluntarily choose whether to participate in this study. If you agree to participate in this study, you need to sign this informed consent, and

agree to our use of your medical information.

2. Cooperate with us for related operations and follow-up.

IV. Who is (not) eligible to participate in the study?

Suitable for participation: 1. Age 18–75 years;

2. Diagnosis of chronic primary insomnia according to the International Classification of Sleep Disorders-third edition (ICSD‑3);

3. Insomnia lasting ≥6 months with a Pittsburgh Sleep Quality Index (PSQI) score >7, and meeting either of the following criteria: a) an inadequate response to both at least one full course of cognitive behavioral therapy for insomnia (CBT-I) and at least one pharmacological treatment for ≥3 months; b) an inadequate response after pharmacological treatment with at least two types of medications with distinct mechanisms of action for ≥3 months;

4. Voluntary participation in this study and signed informed consent;

5. Agreeing not to take other pharmacological or nonpharmacological treatment during the whole trial.

Not suitable for participation: 1. Craniocerebral or scalp injury;

2. Combine severe neurological or mental disorders;

3. Doing night shift work;

4. Prior exposure to electrocon vulsive therapy, transcranial magnetic stimulation, or transcranial current stimulation；

5. With any metal implants in the body, claustrophobia, or any other reasons that would prevent an MRI

6. Being pregnant or lactating;

7. Known hypersensitivity to trial-related medications;

8. History of drug or alcohol abuse/dependence；

9. Participation in concurrent clinical trials.

V. Benefits that may be brought to you by participating in this study

1. Medical Expense Waivers: During the study, the costs of investigational drugs, devices, and related examinations used will typically be borne by the research team, and patients do not need to make additional payments.

2. Access to the Latest Treatment Opportunities: If assigned to the combination therapy group, patients may gain early access to new treatments not yet widely available on the market. This offers a chance to achieve better treatment outcomes, which may include improved sleep quality, shortened sleep latency, increased proportion of deep sleep, and alleviated mood disorders such as anxiety and depression.

3. Attention from Professional Medical Teams: Throughout the study, patients will receive close monitoring and care from a team of authoritative experts. The frequency of examinations and follow-ups may be higher than that of conventional treatment, helping to promptly detect and address changes in the patient's condition.

4. Contributing to Medical Progress: Patients' participation data will provide important information for disease research, helping to advance medical progress and benefit future patients.

VI. Possible adverse reactions, risks, prevention and treatment measures of participating in this study

Potential adverse events (AEs) related to electrical stimulation, including skin redness, pruritus, headache, epileptic seizure, hypomanic, and common adverse events such as dizziness, or nausea, will be closely monitored. In the event of an AE, the participant will receive immediate clinical assessment and necessary medical care. For mild AEs (e.g., minor discomfort), local symptomatic treatment will be provided. For moderate or severe AEs (e.g., syncope, severe pain), the intervention will be paused or terminated immediately, and the participant will be closely monitored by qualified medical staff until full recovery. Emergency procedures will be in place to always ensure patient safety. All AEs will be systematically documented in the Case Report Form (CRF) with a comprehensive assessment of their causal relationships.

VII. Description of expenses

1. This research will not impose any additional financial burden on the participants.

2. All related costs involved in this study, such as clinical procedures, examinations, and medications, are waived. In case of any trial-related injuries, corresponding treatment and compensation will be provided in accordance with national regulations.

VIII. Your Rights

Participation in the study is entirely voluntary. You may decline to participate in the study, or withdraw from the study without reason at any time during the study, without affecting your relationship with your doctor, or the loss of medical or other benefits to you. If you have any questions about this study or during the study, please contact Yudong Wang, Investigator of this study at 17398378680.

IX. Confidentiality of your personal information

Your medical records (including research records and physical and chemical

examination reports, etc.) will be kept in the hospital as required. Except for relevant personnel such as researchers, ethics committees, supervisors, inspectors, and drug administration departments, other personnel not related to the study have no right to access your medical records without permission. Your personal information will not be disclosed in the public report of the results of this study. We will make every effort to protect the privacy of your personal medical information to the extent permitted.

X. Termination of study participation

Your participation in the study may be terminated for the following reasons:

1. You did not follow the study doctor's orders.

2. You have a serious condition that may require treatment.

3. The study doctor believes that terminating the study is in the best interest of your health.

4. You withdraw your consent XI. Ethics Committee

This study has been reported to the Ethics Committee of the Affiliated Hospital of Yangzhou University, and was approved after review by the committee. During the study process, the Ethics Committee of the Affiliated Hospital of Yangzhou University can be contacted for ethics and rights issues.

Tel: +86 514 8031 9670 Email Address: 15950682071@163.com


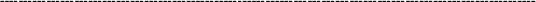


**Researcher statement:**

I confirm that I have explained to the subject the details of the study, including its rights and possible benefits and risks, answered the subject's questions, and the subject stated that he understood my explanations and explanations. I have provided a signed copy of the informed consent to the subject.

Investigator Signature: Investigator Contact Number:

Date of conversation:

**Subject statement:**

I confirm that I have read the informed consent for this study, and the researcher has explained the relevant contents to me in detail, answered my questions, and made me understand the terms and conditions therein. I also confirm that if I do not participate in this study or withdraw at any time during the study, my diagnosis and treatment activities and doctor-patient relationship will not be affected, or other interests will be harmed. I understand that the public reporting of the results of this study will not disclose my personal information. I have had plenty of time to think about it. After careful consideration, I have decided to accept the treatment (research) approach in this study and agree to use my relevant research data and information for public reporting related to the results of this study.

Subject signature: Signature date:

Subject's contact phone number:
